# Cross-Dataset Genomic Proximity and Functional Association of FOXA1 and TET2 at Hypomethylated Regulatory Regions in Colorectal Cancer

**DOI:** 10.3390/cancers18193110

**Published:** 2026-09-25

**Authors:** Donglu Lyu, Beile Lyu, Qingqing Qin, Chengtian Gao, Jie Hu, Fengyang Cao, Heying Luo, Jun Ren, Qiyuan Li, Ying Zhou, Han Jiang

**Affiliations:** 1 Department of Thyroid Surgery, The First Affiliated Hospital of Xiamen University, School of Medicine, Xiamen University, Xiamen 361003, China; 24520241154780@stu.xmu.edu.cn; 2 National Institute for Data Science in Health and Medicine, School of Medicine, Xiamen University, Xiamen 361102, China; qinqq0627@xmu.edu.cn (Q.Q.); hujie2000@stu.xmu.edu.cn (J.H.); 24520200156760@stu.xmu.edu.cn (F.C.); luoheying@stu.xmu.edu.cn (H.L.); renjunn@xmu.edu.cn (J.R.); qiyuan.li@xmu.edu.cn (Q.L.); 3 State Key Laboratory of Cellular Stress Biology, School of Life Sciences, Xiamen University, Xiamen 361100, China; 21620230157115@stu.xmu.edu.cn; 4 Department of Neurosurgery, The First Affiliated Hospital of Xiamen University, Xiamen 361003, China; 24520220157338@stu.xmu.edu.cn; 5 Department of Pediatrics, School of Medicine, Xiamen University, Xiamen 361102, China; 6 Department of Hematology, School of Medicine, Xiamen University, Xiamen 361102, China

**Keywords:** colorectal cancer, FOXA1, TET2, DNA methylation

## Abstract

Background/Objectives: Aberrant DNA methylation plays a critical role in the development and progression of colorectal cancer (CRC). However, how pioneer transcription factors are associated with DNA demethylases at cis-regulatory elements and influence tumor-associated transcriptional programs remains poorly understood. This study investigated the functional relationship between FOXA1 and TET2, an enzyme that converts 5-methylcytosine (5mC) to 5-hydroxymethylcytosine (5hmC), and its association with epigenetic features and malignant phenotypes in CRC. Methods: Public DNA methylation, ChIP-seq, and transcriptomic datasets were integrated with genome-wide genomic-proximity analyses. The association between FOXA1 and TET2 was evaluated by co-immunoprecipitation. FOXA1 and TET2 were individually or simultaneously depleted in HCT116 and SW480 cells, followed by proliferation and invasion assays. In addition, RNA-seq profiles were generated from control, FOXA1-KD, TET2-KD, and Double-KD HCT116 cells to characterize the resulting transcriptional changes. Results: CRC-associated hypomethylated regions showed preferential enrichment of FOXA1- and TET2-associated ChIP-seq signals. At a 500 bp summit-to-summit threshold, FOXA1–TET2 genomic proximity was 30.7-fold higher than expected under chromosome-wise circular permutation (empirical *p* = 0.000999). FOXA1/TET2-associated loci also showed lower methylation than FOXA1-only loci in TCGA-COAD tumors (median within-patient Δβ = −0.051; paired Wilcoxon *p* = 5.86 × 10−50), and FOXA1 and TET2 expression was positively correlated in TCGA-COAD tumors (Spearman’s ρ = 0.40 and *p* = 8.5 × 10−10). Co-immunoprecipitation supported the presence of FOXA1 and TET2 within the same protein complex but did not establish direct physical binding. FOXA1 depletion reduced cell-growth-associated and invasion-associated readouts in both CRC cell lines, whereas TET2 depletion increased growth-associated readouts and showed cell-line-dependent effects on invasion. Combined FOXA1/TET2 depletion produced an intermediate growth phenotype under the conditions examined. RNA-seq revealed broadly concordant transcriptome-wide changes among the knockdown conditions, with pairwise log2 fold-change correlations ranging from r = 0.835 to 0.920, together with shared and comparison-specific transcriptional responses.

## Simple Summary

Colorectal cancer develops through genetic changes and changes in gene regulation. DNA methylation can alter gene activity. FOXA1 helps make DNA accessible, whereas TET2 converts 5-methylcytosine (5mC) to 5-hydroxymethylcytosine (5hmC), a step in DNA demethylation. We examined whether FOXA1 and TET2 are functionally associated in colorectal cancer. Conserved cancer-associated hypomethylated regions were enriched at FOXA1-associated sites and showed local TET2 enrichment. FOXA1- and TET2-associated genomic regions were non-randomly close, particularly near promoters and transcription start sites. Co-immunoprecipitation suggested that the proteins occur in the same protein complex. FOXA1 depletion reduced colorectal cancer cell growth and invasion, whereas combined FOXA1/TET2 depletion partially reversed the effects of FOXA1 loss in selected assays, supporting a phenotypic rescue. RNA sequencing showed partly similar changes in gene activity together with changes associated with each protein. Together, these findings support a context-dependent functional relationship between FOXA1 and TET2 rather than a simple linear pathway.

## 1. Introduction

According to GLOBOCAN 2020 estimates, colorectal cancer (CRC), one of the most common gastrointestinal malignancies, ranked third in cancer incidence and second in cancer-related mortality worldwide and remains a major contributor to cancer morbidity and mortality in the United States [1,2]. Although surgery, chemotherapy, targeted therapy, and immunotherapy have advanced, tumor heterogeneity, therapeutic resistance, and metastasis remain major barriers to long-term survival in patients with CRC [3,4,5,6]. Further elucidation of the molecular mechanisms underlying CRC development and progression is therefore needed to identify new regulatory targets.

In addition to genetic alterations, epigenetic reprogramming is an important molecular feature of CRC. Aberrant DNA methylation can be manifested as hypermethylated differentially methylated positions (Hyper-DMPs) and hypomethylated differentially methylated positions (Hypo-DMPs), thereby affecting chromatin states, cis-regulatory element activity, and downstream gene expression [7,8]. These differentially methylated positions are often enriched in specific genomic regions, suggesting that their distribution may be associated with the activities of transcription factors and epigenetic regulators. Previous studies have characterized FOXA1-regulated transcriptional networks in CRC and identified FOXA1-associated hypomethylation signatures in colon adenocarcinoma. In our previous pan-cancer methylation study, Hypo-MS4 was identified as a hypomethylation signature associated with FOXA1-binding regions, whereas conserved Hypo-DMPs represented individual CpG sites identified across cancer types. TET2 signals were also enriched near conserved Hypo-DMPs based on ChIP-seq data from MCF-7 cells, and related DNA methylation signatures were associated with distant metastasis and immunotherapy responses. Although the TET2 data were derived from breast cancer rather than CRC cells, these findings provided a rationale for investigating the relationship between FOXA1 and TET2 in CRC [9,10,11].

Functionally, FOXA1 is a pioneer transcription factor that can recognize nucleosomal DNA and promote local chromatin accessibility. In lung adenocarcinoma models, FOXA1/2-dependent epigenomic reprogramming has been shown to facilitate TET2/3 recruitment and DNA demethylation, although whether a similar mechanism operates in CRC remains unclear. More broadly, metabolic reprogramming and epigenetic plasticity are important features of cancer biology [12,13,14,15]. In contrast, TET2, as a TET-family enzyme, contributes to DNA demethylation through 5-methylcytosine oxidation; pioneer factors can facilitate access to compacted chromatin, whereas H3K27 demethylases remove methyl marks from histone H3 lysine 27, representing a mechanistically distinct form of epigenetic regulation [16,17,18]. Therefore, FOXA1 and TET2 may be functionally complementary: FOXA1 may contribute to genomic site selection and chromatin accessibility, whereas TET2 may contribute to the regulation of DNA methylation states at the corresponding regions. However, the present study evaluates cross-dataset genomic proximity, protein–complex association, transcriptional responses, and cellular phenotypes rather than direct co-occupancy, directional recruitment, or catalytic coupling.

To investigate this relationship, we integrated DNA methylation, chromatin immunoprecipitation sequencing (ChIP-seq), and transcriptomic datasets with protein–complex association assays, gene knockdown, and cellular functional experiments. Conserved cancer-associated Hypo-DMPs showed enrichment of FOXA1- and TET2-associated signals. FOXA1- and TET2-associated regions also exhibited non-random cross-dataset genomic proximity, particularly near promoters and transcription start sites (TSSs), while co-immunoprecipitation supported the presence of FOXA1 and TET2 within the same protein complex in CRC cells but did not establish direct binding. Functional assays and RNA sequencing (RNA-seq) were further used to examine the shared and context-dependent consequences of FOXA1 and TET2 depletion.

## 2. Materials and Methods

### 2.1. Cell Lines and Cell Culture

The human colorectal cancer cell lines SW480 (RRID: CVCL_0546) and HCT116 (RRID: CVCL_0291) were purchased from the American Type Culture Collection (ATCC) and maintained in our laboratory. Cells were cultured in DMEM supplemented with 10% fetal bovine serum (FBS) at 37 °C in a humidified incubator containing 5% CO_2_.

### 2.2. RNA Extraction and qRT-PCR

Total RNA was extracted using the TRIzol reagent, and cDNA was synthesized using the All-in-One 5× RT MasterMix with gDNA removal (Cat. No. G592; Applied Biological Materials, Richmond, BC, Canada). Quantitative real-time PCR was performed using the SYBR Green qPCR Master Mix on a CFX96 Touch Real-Time PCR Detection System (Bio-Rad, Hercules, CA, USA). GAPDH was used as the internal reference gene, and relative gene expression was calculated using the 2^−ΔΔCt^ method [19]. Primer sequences are listed in Supplementary Table S1.

### 2.3. Establishment of Stable Knockdown Cell Lines

shRNA expression plasmids targeting FOXA1 or TET2, together with a non-targeting negative-control shRNA plasmid, were provided by Miaoling Biotechnology Co., Ltd. (Wuhan, China). The target shRNA plasmids and lentiviral packaging plasmids were co-transfected into HEK293T cells using PEI 40K. Viral supernatants were collected and filtered 48 h after transfection. Target cells were infected with the lentiviral supernatants and subjected to antibiotic selection to establish stable knockdown cell lines. The shRNA sequences are listed in Supplementary Table S2.

### 2.4. Western Blotting

Total protein was extracted using the RIPA lysis buffer supplemented with protease and phosphatase inhibitors. After determination of protein concentrations, 20 μg of protein from each sample was loaded per lane and separated by 8% SDS-PAGE. Proteins were subsequently transferred onto 0.45 μm PVDF membranes at a constant current of 300 mA. The membranes were blocked with 5% non-fat milk in TBST for 1 h at room temperature and then incubated with primary antibodies overnight at 4 °C, followed by incubation with the corresponding HRP-conjugated secondary antibodies for 1 h at room temperature. Protein signals were detected using a chemiluminescent substrate. Antibody information is provided in Supplementary Table S3.

### 2.5. CCK-8 Cell Proliferation Assay

Cells were seeded into 96-well plates at a density of 1 × 10^4^ cells per well, with four replicate wells per group. After incubation for 0, 24, 48, or 72 h, 10 μL of the CCK-8 reagent was added to each well, followed by incubation for 1 h at 37 °C in a humidified atmosphere containing 5% CO_2_. Absorbance at 450 nm was measured using a microplate reader to evaluate cell proliferation. The experiment was repeated independently at least three times.

### 2.6. Transwell Cell Invasion Assay

Cell invasion was assessed using Transwell chambers precoated with Matrigel. Matrigel was diluted 1:8 with serum-free DMEM, and 60 μL of the diluted Matrigel was added to each upper chamber and incubated at 37 °C for 1 h. Cells from each group were harvested, counted, and resuspended in serum-free DMEM. A total of 2.5 × 10^5^ cells were added to each upper chamber, while 600 μL of the complete medium containing 20% FBS was added to the lower chamber. Cells were incubated for either 24 or 48 h under otherwise identical assay conditions. At the indicated time point and after incubation for 48 h, cells remaining on the upper surface of the membrane were removed. Cells that had invaded through the membrane were fixed with 4% paraformaldehyde and stained with 0.1% crystal violet. Three randomly selected microscopic fields per chamber were photographed, and the invaded cells were counted. The 24 h and 48 h Transwell assays were performed as separate experiments using equal starting cell numbers across the experimental groups. In parallel, equal numbers of cells from each experimental group were seeded in conventional 24-well plates and cultured for 24 h. Cells were then fixed, stained with DAPI, and imaged under identical conditions, and DAPI-positive nuclei were counted to assess relative cell accumulation during the same 24 h interval.

### 2.7. Co-Immunoprecipitation Assay

#### 2.7.1. Exogenous Co-Immunoprecipitation

The HA-tagged FOXA1 expression plasmid was transiently transfected into HCT116 and SW480 cells. An HA-tagged empty-vector control was processed in parallel in HCT116 cells to assess nonspecific association with the HA tag or the anti-HA immunoprecipitation system. Cell lysates were collected and incubated with Protein A/G magnetic beads conjugated to a rabbit anti-HA antibody for 2 h at 4 °C. Normal rabbit IgG was used as the negative control. After washing with a lysis buffer, the beads were boiled in an SDS sample buffer to elute the bound proteins, which were subsequently analyzed by Western blotting for HA-FOXA1 and TET2. Input lysates were analyzed in parallel.

#### 2.7.2. Endogenous Co-Immunoprecipitation

Endogenous Co-IP was performed in both HCT116 and SW480 cells. For FOXA1 immunoprecipitation, cell lysates were incubated with Protein A/G magnetic beads conjugated to an anti-FOXA1 antibody for 2 h at 4 °C, and the immunoprecipitated fractions were analyzed by Western blotting for FOXA1 and TET2. For reciprocal Co-IP, TET2 was immunoprecipitated using an anti-TET2 antibody, and the precipitated fractions were analyzed for TET2 and FOXA1. Corresponding IgG controls and input lysates were included in each experiment. After washing, the bound proteins were eluted with an SDS sample buffer and subjected to Western blot analysis. These experiments were performed to assess whether endogenous FOXA1 and TET2 were associated within the same protein complex in both CRC cell models.

### 2.8. Public Database Data Sources

FOXA1 ChIP-seq data from LS180 cells were obtained from the GEO dataset GSE140533, whereas TET2 ChIP-seq data from HCT116 and HT29 cells were obtained from GSE261016. Illumina HumanMethylation450 BeadChip (HM450/450K) DNA methylation data, RNA-seq expression data, and clinical information for the TCGA Colon Adenocarcinoma cohort (TCGA-COAD) were downloaded from the GDC Data Portal. The Hyper-DMP and Hypo-DMP sets used for Figure 1 were obtained from our previously published pan-cancer DNA methylation analysis [10]. Probe-level preprocessing followed the same published HM450 workflow. Starting from 485,577 probes, probes overlapping SNP loci, probes located on chromosome X or Y, and probes with >10% missing values were removed, leaving 381,900 probes (78.65%), with 103,677 probes (21.35%) excluded. The remaining missing beta values were imputed using the across-sample median. Conserved DMPs were derived from 569 matched tumor-normal pairs across nine cancer types using 1000 permutation tests. Probes with *p* < 0.001 were first designated poised DMPs, and median Δβ values across matched pairs were subsequently calculated. Final conserved Hyper-DMPs and Hypo-DMPs were selected using study-specific Δβ thresholds of +0.175 and −0.145, respectively [10].The same QC-filtered HM450 probe set was used for subsequent methylation analyses. The 450K probes were annotated using the Bioconductor package IlluminaHumanMethylation450kanno.ilmn12.

For patient-level integrative methylation analyses, the same QC-filtered HM450 probe set was used, and TCGA-COAD HM450 β-value data were restricted to primary tumor samples (TCGA sample code 01). When multiple aliquots were available for the same patient, β-values were averaged to generate a single patient-level methylation profile. HM450 CpGs were annotated using an hg38/GRCh38 manifest containing genomic coordinates, the CpG-island context, and gene-regulatory annotations. For cross-dataset ChIP-seq integration, the FOXA1 and TET2 datasets were analyzed as independent reference profiles and were not pooled as raw read-count matrices or treated as biological replicates from a single experiment. Therefore, no cross-dataset batch-correction algorithm was applied. For patient-level methylation analyses, FOXA1/TET2-associated and FOXA1-only CpGs were compared within shared CpG-island and gene-regulatory strata to reduce confounding by genomic-context composition. Reference FOXA1 and TET2 ChIP-seq signal values used in locus-level regression analyses were log-transformed and standardized across genomic loci.

Patient-specific regression models evaluated the association between local methylation and the reference TET2 ChIP-seq signal while adjusting for the FOXA1 ChIP-seq signal and the genomic context. For transcript-level analyses, FOXA1 and TET2 expression values were standardized before fitting models containing the FOXA1 × TET2 interaction term. Genome builds were harmonized within each coordinate-based analysis. The conserved DMP coordinates used for Figure 1 and the corresponding ChIP-seq enrichment analyses were analyzed using hg19/GRCh37 coordinates, whereas the patient-level integrative HM450 analyses were performed using hg38/GRCh38 coordinates. For analyses performed in hg19/GRCh37, peak files originally provided in hg38/GRCh38 were converted to hg19/GRCh37 using the UCSC LiftOver tool before window-based, overlap, proximity, and genomic annotation analyses. Patient-level integrative HM450 analyses were performed using hg38/GRCh38 coordinates, and all CpG and ChIP-seq genomic coordinates used in these analyses were harmonized to hg38/GRCh38 before coordinate-based calculations.

Bulk RNA-seq reads generated in this study were aligned to GRCh38 and analyzed at the gene-expression level; these gene-level expression matrices were not directly overlapped with ChIP-seq genomic intervals and therefore did not require coordinate conversion. For the Figure 2 pathway analysis, FOXA1/TET2-associated genomic regions were annotated to genes using the Bioconductor package ChIPseeker. Peaks located within ±3 kb of a transcription start site were assigned to the corresponding promoter-associated gene; peaks located within gene bodies were assigned to the host gene; and intergenic peaks were assigned to the nearest gene.

### 2.9. Bulk RNA-Seq and Data Preprocessing

HCT116 cells were used for the RNA-seq experiment and were subjected to transient knockdown under four conditions: control, FOXA1 knockdown (KD-FOXA1), TET2 knockdown (KD-TET2), and combined FOXA1/TET2 knockdown (Double-KD). Cells were collected 48 h after perturbation, and total RNA was extracted for library construction. These transiently perturbed RNA-seq samples were distinct from the antibiotic-selected stable knockdown populations described in Section 2.3 and used for RT–qPCR, Western blotting, and functional assays. Paired-end sequencing with a read length of 150 bp (PE150) was performed on the DNBSEQ-T7 platform. After quality control, clean reads were aligned to the human GRCh38 reference genome using STAR. GENCODE v44 was used for gene annotation and quantification. Gene-level raw read counts were generated using the GeneCounts mode implemented in STAR based on GENCODE v44 annotations and analyzed with DESeq2 using median-of-ratios normalization. KD-FOXA1, KD-TET2, and Double-KD were each compared with the control group, and genes with an absolute log_2_ fold change >0.5 and a Benjamini–Hochberg-adjusted *p*-value < 0.05 were considered differentially expressed.

### 2.10. Statistical Analysis

Data are presented as the mean ± standard deviation. Comparisons between two groups were performed using a two-tailed *t*-test. Comparisons among multiple groups were performed using one-way or two-way analysis of variance, followed by appropriate corrections for multiple comparisons. Statistical analyses were conducted using GraphPad Prism 10.1, and *p* < 0.05 was considered statistically significant.

## 3. Results

### 3.1. TET2-Associated Signals Are Enriched near Conserved Hypo-DMPs and Show Non-Random Genomic Proximity to FOXA1-Associated Regions

Given the previously identified association between Hypo-MS4 regions and FOXA1-binding regions, we next investigated whether TET2-associated ChIP-seq signals were enriched near conserved hypomethylated regions and whether FOXA1- and TET2-associated genomic regions exhibited non-random spatial proximity [10,11,20]. To assess the association between TET2 chromatin occupancy and COAD-associated differential methylation, we integrated FOXA1 and TET2 ChIP-seq profiles derived from colorectal cancer cell models with the conserved HM450 Hyper-DMP and Hypo-DMP sets defined in our previously published pan-cancer methylation analysis [10]. FOXA1 and TET2 ChIP-seq signals were then profiled within ±3 kb of each conserved DMP probe. Compared with Hyper-DMPs, Hypo-DMPs showed stronger central enrichment of both FOXA1 and TET2 ChIP-seq signals, with the highest signal intensity observed around the center of conserved Hypo-DMP regions (Figure 1A). These findings suggest that conserved hypomethylated regions are preferentially associated with FOXA1- and TET2-enriched genomic contexts. In 480 tumor samples from TCGA-COAD, FOXA1 expression was also significantly and positively correlated with TET2 expression (Spearman’s R = 0.40 and *p* = 8.5 × 10^−10^; Figure 1B).

We next examined the genomic distributions of FOXA1 and TET2 binding. Metagene profiling across transcription start sites (TSSs), normalized gene bodies, and transcription termination sites (TTSs) showed that both FOXA1 and TET2 were preferentially enriched near TSSs, whereas their signals across gene bodies and TTS-proximal regions were relatively lower or more dispersed (Figure 1C). Higher-resolution analysis within ±1 kb of TSSs further confirmed local enrichment of both FOXA1 and TET2 around transcription initiation regions (Figure 1D), rather than serving as an independent validation of the metagene result. Based on the genomic coordinate relationships between FOXA1 and TET2 peaks, we further examined coordinate-defined FOXA1/TET2-associated regions. These sites were predominantly located in promoter regions, although smaller subsets were also distributed in intronic and distal intergenic regions (Figure 1E). Analysis of their distances to the nearest TSS showed that the FOXA1/TET2-associated regions were strongly biased toward promoter- and TSS-proximal regions (Figure 1F). Thus, cross-dataset FOXA1-TET2 genomic proximity is concentrated in promoter/TSS-associated contexts, but this distribution alone does not establish direct regulation of transcription initiation. Permutation analysis further showed that the observed spatial proximity between FOXA1 and TET2 exceeded that expected under the Figure 1G positional null. FOXA1 sites located near TET2 peaks were significantly enriched at the genome-wide level (observed = 4249, expected = 403.1, fold enrichment = 10.54, and empirical *p* = 5.00 × 10^−4^; Figure 1G). This spatial enrichment was even stronger within promoter and TSS ±2 kb regions (observed = 2178, expected = 72.7, fold enrichment = 29.96, and empirical *p* = 5.00 × 10^−4^; Figure 1G). These results indicate that the spatial proximity between FOXA1 and TET2 is unlikely to reflect a random genomic distribution and is particularly pronounced in promoter- and TSS-proximal regions.

To determine whether the coordinate-defined co-localized regions were supported by corresponding ChIP-seq signals, we centered the analysis on FOXA1 sites overlapping TET2 peaks and examined FOXA1 and TET2 signal enrichment. FOXA1 signals formed a pronounced peak at the binding-site center, whereas TET2 signals also showed local enrichment around the same set of FOXA1 sites (Figure 1H), supporting their local co-enrichment. Finally, FOXA1 peak centers were stratified according to their distance to the nearest TET2 peak. FOXA1 sites located within 500 bp of a TET2 peak showed stronger and more concentrated TET2 signals around the FOXA1 peak center than FOXA1 sites located 500–2000 bp from the nearest TET2 peak (Figure 1I). This distance-dependent pattern suggests that closer genomic proximity between FOXA1 and TET2 peaks is associated with more pronounced local co-enrichment of their ChIP-seq signals.

### 3.2. Genes Associated with FOXA1/TET2-Associated Genomic Regions Are Enriched in Cancer-Related Signaling and Gene-Expression Pathways

To investigate the potential biological functions associated with FOXA1–TET2 co-localized regions, the FOXA1/TET2-associated regions were annotated to nearby or associated genes, and the resulting gene set was subjected to pathway enrichment analysis. Reactome analysis showed that FOXA1–TET2 co-localized genes were enriched primarily in WNT/β-catenin signaling, nuclear receptor signaling, transcriptional regulation, pre-mRNA processing, mRNA splicing, protein translation, and chromatin-associated processes such as histone acetylation (Figure 2A). These findings suggest that FOXA1–TET2 co-localized regions may be associated with multiple levels of gene expression regulation, including transcription, RNA processing, and protein production. To identify potential core molecules within the enriched pathways, we further counted genes that repeatedly occurred across significant Reactome pathways. The recurrent genes included transcriptional and cell-state regulators such as MYC, ATF2, and TLE3; signaling and cell-survival molecules such as AKT2, PTK2, XIAP, YWHAZ, and CALM1/CALM2; protein-homeostasis components such as UBC, PSMB2, and PSMB7; and chromatin-related genes, including H1-0 and several H2A/H2B histone family members (Figure 2B). These recurrent genes may represent candidate regulatory nodes connecting FOXA1–TET2 co-localized regions with cancer-related signaling and gene expression networks. However, whether they are directly regulated by FOXA1 and TET2 requires further validation. Hallmark analysis further showed enrichment of TNFα signaling via NF-κB, the p53 pathway, apoptosis, E2F targets, hypoxia, TGFβ signaling, and MYC targets (Figure 2C). Together, these results indicate that FOXA1–TET2 co-localized genes are mainly associated with cancer-related signaling, cell proliferation, inflammatory and stress responses, and gene expression regulation.

### 3.3. FOXA1/TET2-Associated Genomic Regions Are Preferentially Associated with Local Hypomethylation

To further investigate the relationship between FOXA1, TET2, and DNA methylation, we integrated FOXA1 and TET2 ChIP-seq profiles with TCGA-COAD HM450 methylation data. Chromosome-wise circular permutation analysis revealed a significant non-random genomic association between FOXA1- and TET2-associated regions. At the primary 500 bp summit-to-summit threshold, 2940 FOXA1 summits were located within 500 bp of a TET2 summit, compared with a mean of 95.6 expected under 1000 circular permutations, corresponding to a 30.7-fold enrichment (empirical *p* = 0.000999; Figure 3A). This association remained robust across alternative distance thresholds, with observed-to-expected enrichments of 53.2-, 42.4-, 30.7-, and 19.3-fold at 100, 250, 500, and 1000 bp, respectively (Supplementary Figure S1C). Continuous TET2 ChIP-seq signal profiles centered on FOXA1-associated regions further showed stronger local TET2 enrichment in FOXA1/TET2-associated regions than in FOXA1-only regions (Supplementary Figure S1D). This summit-based definition is distinct from the 2 kb peak-proximity and interval-overlap definitions used in Figure 1; therefore, the Figure 3A count of 2940 and its 1000-permutation *p* = 0.000999 are not expected to match the Figure 1G values. The empirical *p*-value quantifies departure from the specified circular-shift null and should not be interpreted as evidence of FOXA1-dependent TET2 recruitment. Likewise, the 30.7-fold value represents genomic-proximity enrichment rather than a DNA-methylation effect size.

To investigate whether TET2-associated genomic proximity was associated with local DNA methylation patterns, we examined methylation levels according to CpG-to-TET2 summit distance. FOXA1-associated CpGs located closest to the nearest TET2 summits exhibited the lowest methylation levels, whereas methylation progressively increased with increasing distance from TET2 summits (Figure 3B). The strongest hypomethylation was observed within proximal regions surrounding TET2 summits, indicating that reduced DNA methylation preferentially occurred near TET2-associated genomic regions. We next investigated whether FOXA1/TET2-associated loci displayed distinct methylation patterns compared with FOXA1-only loci. Because these genomic groups differed in CpG-island and gene-regulatory-context distributions (Supplementary Figure S1B), methylation levels were compared within shared genomic-context strata to reduce potential confounding effects from baseline genomic composition. Patient-level context-standardized analysis showed that FOXA1/TET2-associated loci exhibited significantly lower methylation than FOXA1-only loci across TCGA-COAD tumors, with a median within-patient methylation difference of Δβ = −0.051 (paired Wilcoxon test, with *p* = 5.86 × 10^−50^; Figure 3C).

A complementary context-matched probe-level analysis confirmed the same direction of association, showing significantly lower methylation at FOXA1/TET2-associated CpGs than at FOXA1-only CpGs (Wilcoxon test, with *p* = 8.76 × 10^−59^; Supplementary Figure S1A). Consistent with the distance-dependent methylation pattern, patient-specific regression models showed that a stronger reference TET2 ChIP-seq signal was associated with lower local DNA methylation after adjustment for the FOXA1 ChIP-seq signal and the genomic context. The adjusted TET2-signal coefficients were predominantly negative, with a median coefficient of −0.0274 (Wilcoxon test, with *p* = 5.86 × 10^−50^; Figure 3D). At the individual-locus level, the standardized log-transformed reference TET2 ChIP-seq signal was inversely associated with mean TCGA-COAD methylation, with loci showing stronger TET2-associated signals preferentially exhibiting lower methylation levels (Supplementary Figure S1H).

The context-stratified probe-level result in Supplementary Figure S1A was used as a sensitivity analysis: it compares probe distributions within shared genomic-context strata but is not a one-to-one paired-probe analysis, and spatial correlation among neighboring CpGs limits interpretation of its nominal *p*-value as independent biological replication. Although Figure 3C,D yielded the same nominal signed-rank *p*-value, they test different patient-level quantities and have distinct effect-size scales (median Δβ versus adjusted TET2-signal coefficients); the identical *p*-values therefore were not interpreted as duplicated effect estimates.

We additionally examined whether transcript abundance could explain the observed locus-specific methylation pattern. FOXA1 and TET2 expression showed a modest positive correlation in the subset of TCGA-COAD primary tumors with matched RNA-seq and HM450 profiles (Spearman’s ρ = 0.274 and *p* = 1.92 × 10^−6^; Supplementary Figure S1G). However, in a continuous model incorporating standardized FOXA1 expression, standardized TET2 expression, and their interaction term, the FOXA1 × TET2 interaction was not significantly associated with methylation differences between FOXA1/TET2-associated and FOXA1-only loci (interaction β = 4 × 10^−4^ and *p* = 0.286; Supplementary Figure S1E). Classification of patients into FOXA1-low/TET2-low, FOXA1-high/TET2-low, FOXA1-low/TET2-high, and FOXA1-high/TET2-high groups provided complementary visualization of expression-associated methylation patterns (Supplementary Figure S1F).

Together, these analyses demonstrate that FOXA1-associated genomic regions characterized by closer TET2 proximity and stronger TET2-associated ChIP-seq signals are preferentially associated with local DNA hypomethylation in TCGA-COAD tumors. Because these analyses are observational and HM450 bisulfite measurements do not distinguish 5mC from 5hmC, they demonstrate an association between FOXA1/TET2-associated genomic features and local hypomethylation but do not establish FOXA1-dependent TET2 recruitment or TET2-mediated 5mC oxidation.

### 3.4. Establishment and Validation of Stable Single and Combined FOXA1/TET2 Knockdown Models in Colorectal Cancer Cells

To further investigate the functional consequences of FOXA1 and TET2 depletion in colorectal cancer cells, we established stable single- and double-knockdown models of FOXA1 and TET2 in HCT116 and SW480 cells. These models provided experimental systems for examining the protein-level association between FOXA1 and TET2 and for assessing the effects of their individual or combined depletion on colorectal cancer cell growth, invasion, and transcriptional programs.

Before generating the stable knockdown cell lines, candidate shRNAs targeting FOXA1 and TET2 were initially screened in HEK293T cells, which have a relatively high transfection efficiency. Based on knockdown efficiency, stability, and experimental reproducibility, shFOXA1-02 and shTET2-01 were selected for subsequent experiments (Supplementary Figure S2). HCT116 and SW480 cells were then subjected to lentiviral transduction and puromycin selection to establish negative-control (NC) cells, stable FOXA1-knockdown (FOXA1 KD) cells, stable TET2-knockdown (TET2 KD) cells, and combined FOXA1/TET2-knockdown (Double-KD) cells. Broad and relatively homogeneous GFP fluorescence was observed in all lentivirus-transduced groups, indicating successful delivery and stable expression of the corresponding shRNA constructs in the selected cell populations (Figure 4A). Following confirmation of successful lentiviral transduction and stable selection, knockdown efficiency was evaluated at both the mRNA and protein levels. RT–qPCR analysis showed that FOXA1 mRNA expression was significantly reduced in the FOXA1 KD and Double-KD groups compared with the NC group in both HCT116 and SW480 cells (both *p* < 0.0001; Figure 4B). Similarly, TET2 mRNA expression was significantly decreased in the TET2 KD and Double-KD groups in both cell lines (TET2 KD, both *p* < 0.0001; Double-KD, both *p* < 0.001; Figure 4B). These results confirm that the selected shRNAs stably and effectively suppressed transcription of their corresponding target genes in two distinct colorectal cancer cell backgrounds.

Western blotting and densitometric analyses were then performed primarily to validate knockdown efficiency at the protein level. In both HCT116 and SW480 cells, FOXA1 protein abundance was markedly reduced following FOXA1 knockdown, whereas TET2 protein abundance was markedly reduced following TET2 knockdown (Figure 4C–F). In the Double-KD groups, the protein abundance of both FOXA1 and TET2 was lower than that in the NC group, although the magnitude of reduction was less pronounced than that observed in the corresponding single-knockdown groups under some conditions. GAPDH levels remained relatively stable across the experimental groups, supporting consistent protein loading. Overall, these results confirmed the successful establishment of stable single- and combined-knockdown models at both the mRNA and protein levels.Additional RT–qPCR and Western blot analyses were performed to examine TET2 expression following FOXA1 knockdown and FOXA1 expression following TET2 knockdown (Supplementary Figure S11).

Cross-factor expression changes were also observed; however, their magnitude and direction were not fully consistent across perturbation settings and independent experiments, particularly for FOXA1 following TET2 depletion. These changes were therefore considered secondary observations and were not interpreted as evidence of a fixed reciprocal regulatory relationship between FOXA1 and TET2.

### 3.5. FOXA1 and TET2 Show Protein-Level Association and Have Distinct Effects on Colorectal Cancer Cell Growth-Associated and Invasion-Associated Phenotypes

Building on the FOXA1–TET2 expression correlation and chromatin co-localization identified in the preceding multi-omics analyses and using the stable knockdown models established in Figure 3, we further examined the potential relationship between FOXA1 and TET2 at the protein level and assessed their effects on colorectal cancer cell phenotypes. To exclude the possibility of nonspecific binding between TET2 and the HA tag or the anti-HA immunoprecipitation system, a control immunoprecipitation was first performed in HCT116 cells expressing an HA-tagged empty vector. TET2 was readily detected in the input lysate but was not enriched in the anti-HA immunoprecipitate, suggesting that TET2 was not nonspecifically captured by the HA tag or the immunoprecipitation system under these experimental conditions (Figure 5A). We next performed exogenous co-immunoprecipitation analysis in both HCT116 and SW480 cells expressing HA-tagged FOXA1.Anti-HA immunoprecipitation efficiently enriched HA–FOXA1, and TET2 was detected in the same immunoprecipitated fraction, whereas no clear corresponding signal was observed in the IgG control (Figure 5B). Reciprocal endogenous co-immunoprecipitation was further performed using anti-TET2 and anti-FOXA1 antibodies in HCT116 and SW480 cells, respectively. FOXA1 and TET2 were detected in the corresponding immunoprecipitated fractions, whereas no evident enrichment was observed in IgG controls (Figure 4C,D). These findings provide forward and reciprocal protein-level evidence supporting an association between FOXA1 and TET2 in the same immunoprecipitated complexes. However, Co-IP alone does not establish direct physical binding between FOXA1 and TET2. We then used the stable FOXA1 and/or TET2 knockdown models to assess their effects on colorectal cancer cell proliferation. CCK-8 assays showed that FOXA1 knockdown was associated with a downward shift in the 450 nm absorbance curves in both HCT116 and SW480 cells, suggesting reduced cell viability or proliferative capacity.

Transwell invasion assays showed a broadly consistent phenotypic pattern. In both HCT116 and SW480 cells, FOXA1 knockdown was associated with a reduction in the number of cells that traversed the Matrigel-coated membrane, suggesting impaired invasive capacity. The effect of TET2 knockdown was cell-line-dependent: TET2 depletion increased invaded-cell numbers in HCT116 cells but showed a different pattern in SW480 cells. Thus, FOXA1 depletion consistently impaired invasion-associated phenotypes, whereas the effect of TET2 depletion was context-dependent across CRC models. Because Transwell invaded-cell counts were analyzed separately from cell accumulation assays, these results were interpreted as changes in invasion-associated readouts rather than direct measurements of proliferation-independent invasion.

### 3.6. FOXA1 and TET2 Knockdown Converge on Shared Stress- and Proliferation-Related Transcriptional Programs

To characterize the transcriptomic changes associated with FOXA1 and TET2 depletion, RNA sequencing was performed in HCT116 cells following FOXA1 knockdown (FOXA1-KD), TET2 knockdown (TET2-KD), or combined FOXA1 and TET2 knockdown (Double-KD). Principal-component and sample-distance analyses separated the knockdown groups from controls and showed within-group clustering (Supplementary Figure S3A,B). Expression profiles of FOXA1, TET2, and AKT2 are shown in Supplementary Figure S3C. These transient-transfection data were analyzed separately from the stable knockdown models used for protein and functional assays. AKT2 was included as an illustrative pathway-associated gene because it was among the recurrent genes identified in the Figure 2B pathway analysis; its display in Supplementary Figure S3C does not establish AKT2 as a direct FOXA1/TET2 target. Notably, TET2 transcript abundance in FOXA1-KD HCT116 cells was approximately threefold higher than in control cells, consistent with the increased TET2 protein abundance observed after FOXA1 depletion and providing transcript-level support for this direction of cross-regulation (Supplementary Figure S3C).

Compared with the control group, FOXA1-KD, TET2-KD, and Double-KD cells exhibited extensive transcriptional alterations. Volcano plots revealed substantial numbers of upregulated and downregulated differentially expressed genes under all three knockdown conditions, with broadly similar overall expression patterns across the comparisons (Figure 6A). Heatmaps of the top 60 differentially expressed genes clearly separated the control samples from the corresponding knockdown samples, whereas biological replicates within each condition exhibited consistent expression patterns (Figure 6B). FOXA1-KD, TET2-KD, and Double-KD also shared substantial numbers of upregulated and downregulated differentially expressed genes (Supplementary Figure S4A–C). Pairwise correlations of transcriptome-wide log_2_ fold-change values were strong and positive across the three knockdown conditions (FOXA1-KD versus TET2-KD, r = 0.835; FOXA1-KD versus Double-KD, r = 0.920; TET2-KD versus Double-KD, r = 0.879; Supplementary Figure S5A). Because all three contrasts used the same control group, these correlations were interpreted descriptively and may partly reflect shared control-related variation rather than independent evidence of a common mechanism. Likewise, directional concordance among threshold-defined shared DEGs (Supplementary Figure S5B,C) was treated as a descriptive property of the selected DEG sets rather than as an independent validation.

Hallmark enrichment analyses identified stress-associated programs, including TNFα signaling via NF-κB, hypoxia, and glycolysis, among upregulated genes in the knockdown comparisons, whereas downregulated genes were enriched for cell-cycle-associated programs, including E2F targets, MYC targets, and the G2M checkpoint (all FDR < 0.05; Figure 6C,D). The shared signature may partly reflect a common perturbation or transfection-associated stress response and should not be interpreted as proof that FOXA1 and TET2 act through a single mechanism.

Despite broad similarity in the transcriptomic responses, some genes met the differential-expression threshold in only one single-knockdown comparison. The FOXA1-KD-only threshold set contained 367 upregulated and 326 downregulated genes and was enriched for TGF-β signaling, cytoskeletal regulation, and ribosome biogenesis (Supplementary Figure S6). The TET2-KD-only threshold set contained 772 upregulated and 726 downregulated genes and was enriched for developmental, extracellular-matrix, cell-cycle, and DNA-replication terms (Supplementary Figure S7). Because these sets were defined by comparison-specific significance thresholds rather than a direct FOXA1-KD versus TET2-KD test, they are described as threshold-exclusive gene sets rather than proof of factor-specific regulation, and their sizes were not used to infer the causal basis of the opposing cellular phenotypes.

## 4. Discussion

Colorectal cancer development and progression are driven not only by genetic alterations but also by widespread DNA methylation abnormalities and remodeling of transcriptional networks [21,22]. However, during cancer-associated epigenetic reprogramming, how sequence-specific transcription factors guide DNA demethylases to defined regulatory regions and thereby influence downstream transcriptional programs remains incompletely understood.

In this study, by integrating public ChIP-seq datasets, TCGA-COAD methylation and transcriptomic data, co-immunoprecipitation assays, bulk RNA-seq, and cellular functional experiments, we provide initial evidence for a context-dependent, multilayered regulatory relationship between FOXA1 and TET2 in colorectal cancer. First, FOXA1-bound regions showed a clear spatial association with conserved cancer-associated hypomethylated regions, and a subset of these regions also displayed local TET2-associated ChIP-seq signals. These findings support an association between FOXA1/TET2-associated chromatin features and hypomethylated regulatory regions but do not establish simultaneous occupancy in the same cells or directional recruitment of TET2 by FOXA1. Furthermore, exogenous and reciprocal endogenous co-immunoprecipitation assays indicated that FOXA1 and TET2 may coexist within the same protein complex, providing additional support for their potential molecular association.

Given that FOXA1, as a pioneer transcription factor, has site-selective DNA-binding and chromatin-regulatory capacity and that TET2 is involved in 5mC oxidation and DNA demethylation [12,23,24], one possible model is that FOXA1-bound chromatin regions provide genomic contexts in which TET2 is preferentially enriched. However, the present data do not establish that FOXA1 directly recruits TET2. TET2 protein abundance, chromatin association, and catalytic activity should also not be considered equivalent. Although stronger reference TET2 ChIP-seq signals were associated with lower DNA methylation, TET2 catalytic activity, 5hmC production, and TET2 chromatin occupancy after FOXA1 depletion were not directly measured. Therefore, these observations cannot establish a complete mechanistic pathway in which FOXA1 recruits TET2 to drive DNA demethylation.

RNA-seq analysis revealed substantial shared transcriptional changes following FOXA1 and TET2 knockdown, together with comparison-specific threshold-exclusive gene sets. The shared signature may partly reflect a common cellular perturbation or transfection-associated stress response rather than a single FOXA1/TET2-dependent mechanism. In addition, RNA-seq was performed in transiently transfected HCT116 cells, whereas the protein and functional assays used stable knockdown models; these datasets therefore provide complementary but not directly matched perturbation readouts. Previous studies have shown that acute/transient and prolonged stable depletion can produce different downstream gene-expression responses even when depletion of the targeted factor is comparable [25,26]. In HCT116 cells, for example, Neilsen et al. reported that some expression changes observed after stable shRNA-mediated depletion were absent following transient siRNA-mediated depletion, consistent with the possibility of cellular adaptation to prolonged perturbation [26]. Accordingly, differences between the transient RNA-seq measurements and stable-knockdown protein measurements in the present study should not be interpreted as direct mRNA–protein discrepancies within the same biological state.

Consistent with this distinction, the cross-factor expression responses were not fully symmetrical. FOXA1 depletion was accompanied by increased TET2 expression under the experimental conditions examined, whereas the effect of TET2 depletion on FOXA1 expression was more variable across independent experiments and perturbation settings, with no reproducible directional change being established. We therefore do not interpret these observations as evidence of reciprocal negative regulation between FOXA1 and TET2. Instead, the non-targeted-factor changes are considered context-dependent secondary expression responses that may reflect differences in perturbation duration, cellular adaptation, transcriptional reprogramming, protein regulation, or other indirect effects. Direct regulatory relationships between FOXA1 and TET2 would require dedicated mechanistic experiments and cannot be inferred from the present expression measurements alone.

The phenotypic results further showed distinct and partly opposing effects of the two perturbations: FOXA1 depletion reduced growth-associated and invasion-associated readouts in both HCT116 and SW480 cells, whereas TET2 depletion increased growth-associated readouts and showed cell-line-dependent effects on invasion. Combined FOXA1/TET2 depletion produced context-dependent phenotypes rather than a uniform additive or rescue response. These findings argue against a simple cooperative, additive, or linear upstream–downstream relationship between FOXA1 and TET2.

At the phenotypic level, the growth-enhancing effect of TET2 depletion is consistent with previous CRC evidence supporting a tumor-suppressive role for TET2, whereas its effect on invasion was cell-line-dependent in the present study.

Reduced nuclear localization of TET2 has been associated with lower 5hmC levels, lymph-node invasion, distant metastasis, and poorer survival, whereas nuclear TET2 has been reported to promote DNA demethylation and suppress CRC cell growth [27]. More recent work further demonstrated dynamic cytoplasm–nucleus shuttling of TET2 during CRC progression and prolonged cellular culture, with TET2 redistribution associated with EMT/WNT-related cellular-state changes [28]. These findings suggest that TET2 function in CRC may depend not only on total protein abundance but also on its subcellular distribution. Thus, total cellular TET2 abundance should not necessarily be considered equivalent to nuclear or chromatin-associated TET2 activity. However, because TET2 subcellular localization was not directly measured in the present experiments, these observations provide biological context rather than a direct mechanistic explanation for the variable FOXA1 response following TET2 depletion.

Together, the opposing phenotypic effects of FOXA1 and TET2 depletion further argue against interpreting their genomic and protein-level association as a simple cooperative pro-tumorigenic relationship.

Nevertheless, this study has several limitations. First, the current findings are based primarily on in vitro colorectal cancer cell-line models and retrospective public datasets. The FOXA1 and TET2 ChIP-seq profiles were derived from different colorectal cancer cell models, and chromatin occupancy was not examined in the same experimental background following FOXA1 or TET2 depletion. Therefore, the coordinate-based association should be interpreted as cross-dataset genomic proximity and local signal co-enrichment rather than direct evidence of simultaneous occupancy in the same cells. FOXA1-dependent recruitment of TET2 has consequently not been directly established, and future ChIP-seq or CUT&Tag analyses in matched control and knockdown models will be required to determine occupancy dependence and directionality. Sequence-motif enrichment was also not performed for FOXA1/TET2-associated regions; therefore, the present study does not identify additional candidate sequence-specific co-regulators on the basis of motif analysis.

Second, although reciprocal endogenous co-immunoprecipitation supports the possibility that FOXA1 and TET2 may exist in the same protein complex, Co-IP does not demonstrate a direct physical interaction between the two proteins. Future studies using GST pull-down assays, recombinant protein-binding assays, and interaction-domain mapping will be needed to determine whether FOXA1 and TET2 interact directly and, if so, define the relevant interaction domains.

Third, locus-specific DNA methylation was not directly measured following FOXA1 or TET2 depletion, and 5hmC was not directly quantified in the experimental models. The TCGA-COAD HM450 analysis therefore provides observational patient-level support for local hypomethylation rather than perturbation-based mechanistic validation. Moreover, the 450K array covers only a subset of CpG sites, and conventional bisulfite-based array signals cannot distinguish 5mC from 5hmC. Consequently, the observed hypomethylation cannot be attributed specifically to TET2-mediated 5mC oxidation. Future perturbation-based chromatin analyses together with locus-specific and 5mC/5hmC-specific assays will be required to validate the proposed epigenetic mechanism.

Fourth, the molecular basis for the distinct and partly opposing phenotypic effects of FOXA1 and TET2 depletion remains unresolved. The threshold-exclusive RNA-seq gene sets were defined by comparison-specific significance thresholds rather than by a direct FOXA1-KD versus TET2-KD contrast and therefore should not be interpreted as direct evidence of factor-specific regulation or as the causal basis of the phenotypic differences. Similarly, although FOXA1 depletion was accompanied by increased TET2 expression under the conditions examined, FOXA1 expression following TET2 depletion did not show a reproducible directional response across independent experiments. Thus, the present data do not establish reciprocal transcriptional regulation between FOXA1 and TET2.

Fifth, bulk RNA-seq was performed in transiently transfected HCT116 cells, whereas the protein and functional assays were performed using stable knockdown models in HCT116 and SW480 cells. Thus, the transcriptomic and phenotypic datasets were generated under different perturbation settings, and acute and prolonged depletion can generate different secondary expression responses [25,26]. Therefore, the transcriptomic and protein datasets should be regarded as complementary rather than directly matched measurements of the same perturbation state. Validation using matched RNA and protein measurements obtained from the same stable knockdown populations and at matched time points will be required to determine the reproducibility and temporal characteristics of the cross-factor expression responses. The transcriptional findings also require validation in additional CRC cellular backgrounds.

Sixth, although multiple candidate shRNAs targeting FOXA1 and TET2 were initially screened and the constructs used for downstream experiments were selected on the basis of knockdown efficiency and experimental reproducibility, the key proliferation and invasion phenotypes were not independently replicated using a second sequence-independent shRNA. Therefore, sequence-specific off-target effects cannot be completely excluded. Future studies using additional independent shRNA sequences and/or rescue experiments will be important to confirm the target specificity of the observed phenotypes.

Finally, potential redundancy or compensation among TET-family members, including TET1 and TET3, was not systematically examined, and the in vivo relevance of the FOXA1–TET2 regulatory relationship remains to be determined. Future studies using matched perturbation systems, additional CRC cellular backgrounds, and in vivo models will be required to further establish the biological relevance and mechanistic basis of the FOXA1–TET2 association in colorectal cancer.

## 5. Conclusions

This study investigated the potential epigenetic role of the FOXA1–TET2 regulatory relationship in colorectal cancer. We observed preferential FOXA1- and TET2-associated ChIP-seq signals near conserved hypomethylated regions, together with non-random cross-dataset genomic proximity between FOXA1- and TET2-associated regions. Patient-level TCGA-COAD analyses further showed preferential local hypomethylation at FOXA1/TET2-associated loci. Co-IP assays suggested that FOXA1 and TET2 may coexist within the same protein complex. Functional assays revealed opposing phenotypic effects of FOXA1 and TET2 depletion, whereas bulk RNA-seq showed substantial shared transcriptional responses together with comparison-specific threshold-exclusive gene sets. Overall, this study supports a context-dependent, multilayered FOXA1–TET2 relationship associated with genomic features, DNA methylation states, transcriptional changes, and cellular phenotypes while indicating that FOXA1-dependent TET2 recruitment, TET2 catalytic activity, 5mC/5hmC remodeling, and the molecular basis of the opposing phenotypic effects require further validation.

## Figures and Tables

**Figure 1:**
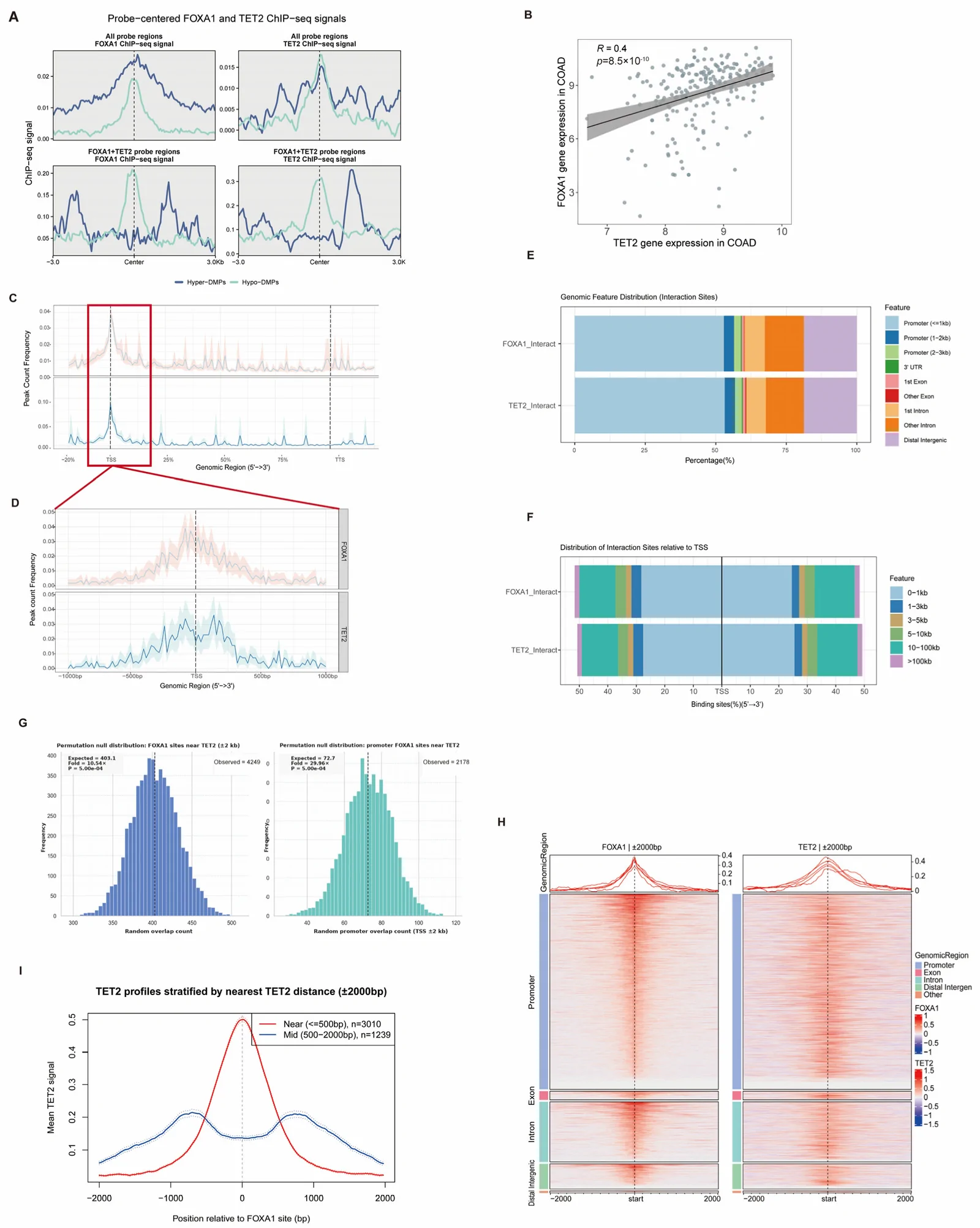
DNA methylation-associated TET2 enrichment and genome-wide spatial association between FOXA1 and TET2. (**A**) FOXA1 and TET2 ChIP-seq signal profiles centered on conserved Hyper-DMPs and Hypo-DMPs within a ±3 kb window. FOXA1 and TET2 signals were evaluated around conserved HM450 DMPs and coordinate-defined FOXA1/TET2-associated regions. (**B**) Correlation analysis between FOXA1 and TET2 expression in TCGA-COAD tumor samples. (**C**) Genome-wide distribution of FOXA1/TET2-associated genomic regions across normalized genomic regions. Dashed lines indicate transcription start sites (TSSs), and the boxed region highlights enrichment near the TSS. (**D**) FOXA1 and TET2 peak-frequency profiles within a ±1 kb window centered on transcription start sites. (**E**) Genomic annotation of FOXA1/TET2-associated regions. (**F**) Distribution of FOXA1/TET2-associated regions relative to transcription start sites. (**G**) Permutation analysis of FOXA1–TET2 spatial proximity genome-wide and within promoter regions. (**H**) Heatmaps and average profiles of FOXA1 and TET2 ChIP-seq signals around FOXA1/TET2-associated regions. (**I**) TET2 signal profiles around FOXA1 sites stratified by distance to the nearest TET2 peak.

**Figure 2:**
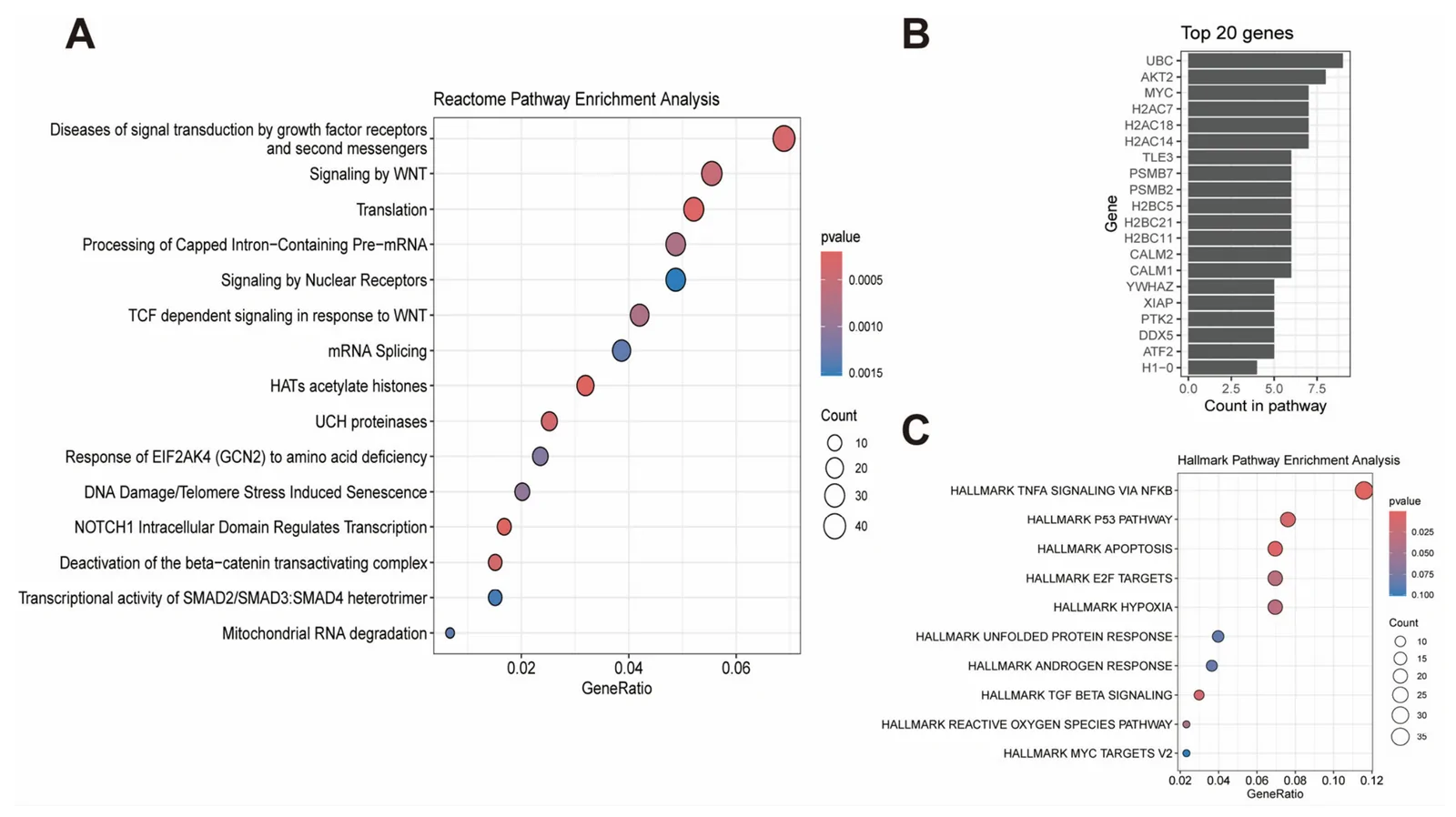
Functional pathway characteristics of genes associated with FOXA1/TET2-associated regions. (**A**) Reactome pathway enrichment analysis of genes associated with FOXA1/TET2-associated regions. (**B**) Top 20 recurrent genes appearing across significantly enriched Reactome pathways. (**C**) Hallmark gene set enrichment analysis of genes associated with FOXA1/TET2-associated regions.

**Figure 3:**
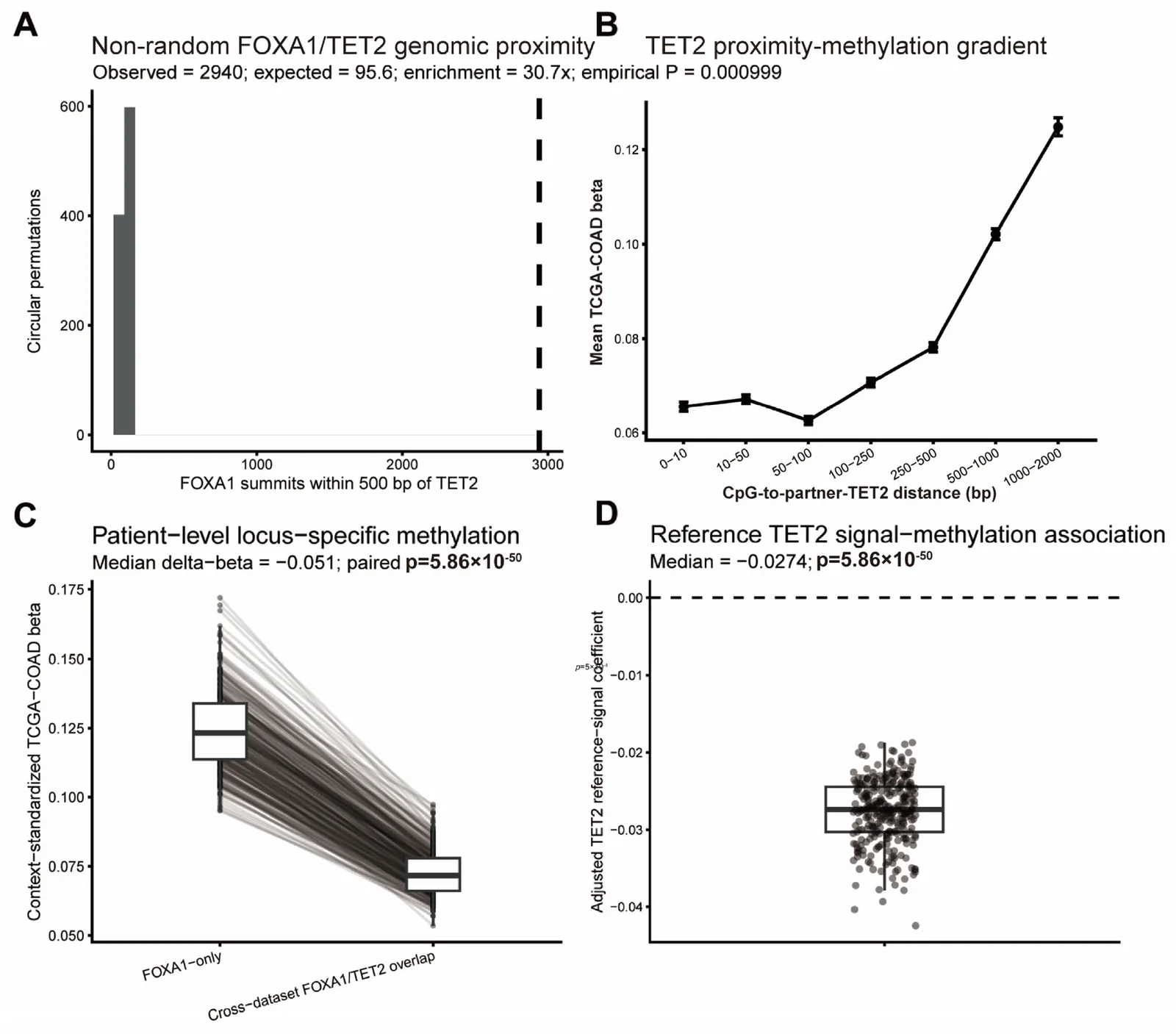
FOXA1/TET2-associated genomic regions are preferentially associated with local hypomethylation in colorectal cancer. (**A**) Chromosome-wise circular permutation analysis of FOXA1/TET2 genomic proximity using a 500 bp summit-to-summit distance threshold. (**B**) Distance-dependent association between CpG methylation levels and proximity to TET2 summits. Mean TCGA-COAD methylation levels were calculated across increasing CpG-to-TET2 distance intervals. (**C**) Patient-level context-standardized comparison of DNA methylation between FOXA1/TET2-associated and FOXA1-only loci in TCGA-COAD tumors. (**D**) Patient-level association between the reference TET2 ChIP-seq signal and local DNA methylation after adjustment for the FOXA1 ChIP-seq signal and the genomic context.

**Figure 4:**
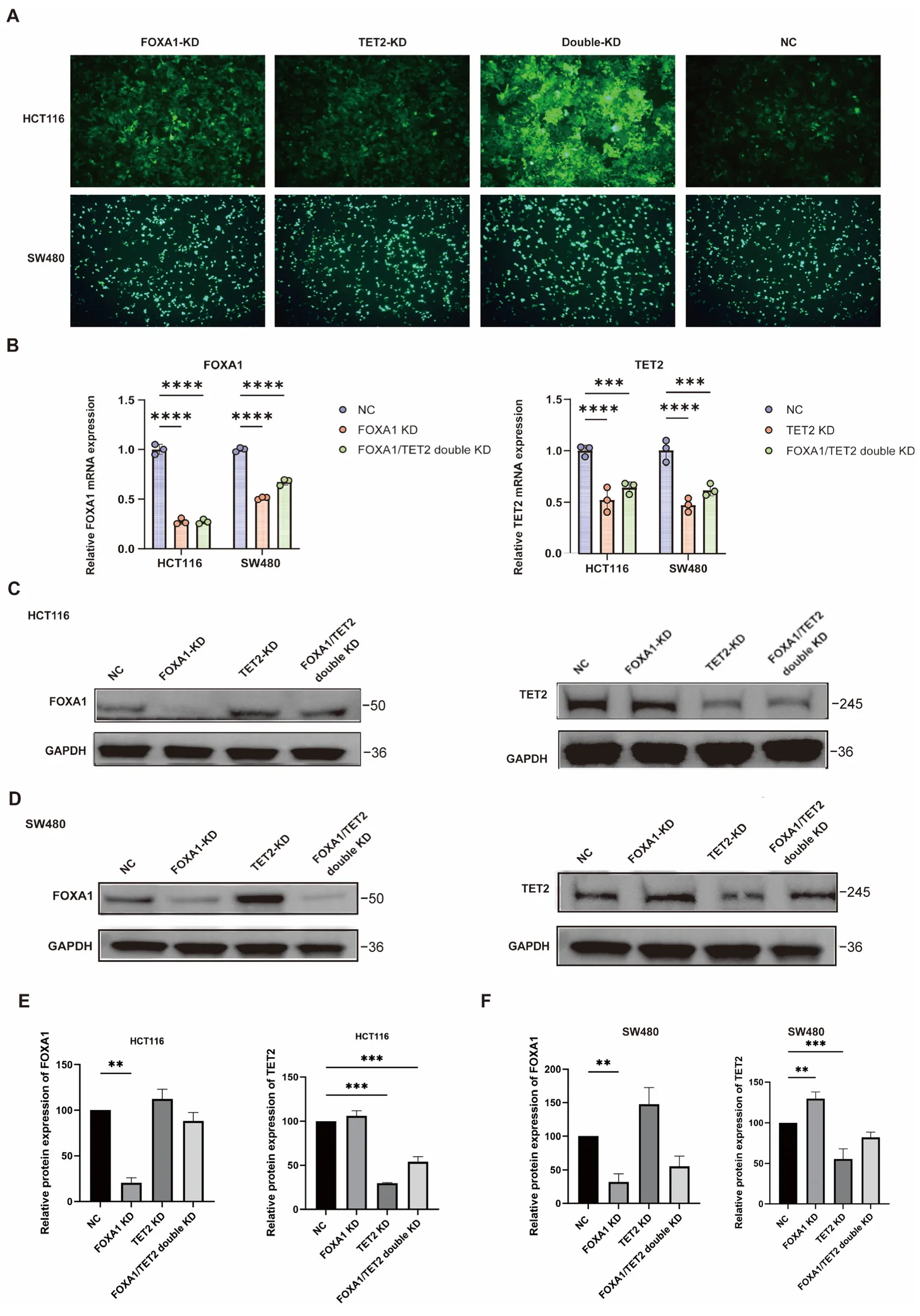
Establishment and validation of stable FOXA1 and/or TET2 knockdown models in HCT116 and SW480 cells. (**A**) Representative GFP fluorescence images of the indicated stable knockdown cells. (**B**) Relative mRNA expression levels of FOXA1 and TET2 in HCT116 and SW480 cells (mean ± SD, *n* = 3; *** *p* < 0.001 and **** *p* < 0.0001). (**C**) Representative Western blots showing FOXA1 and TET2 protein expression in HCT116 cells (WB original images are included in Supplementary Figure S8). (**D**) Representative Western blots showing FOXA1 and TET2 protein expression in SW480 cells (WB original images are included in Supplementary Figure S9). (**E**) Densitometric quantification of FOXA1 and TET2 protein expression in HCT116 cells (mean ± SD, *n* = 3; and ** *p* < 0.01, *** *p* < 0.001). (**F**) Densitometric quantification of FOXA1 and TET2 protein expression in SW480 cells (mean ± SD, *n* = 3; ** *p* < 0.01, *** *p* < 0.001).

**Figure 5:**
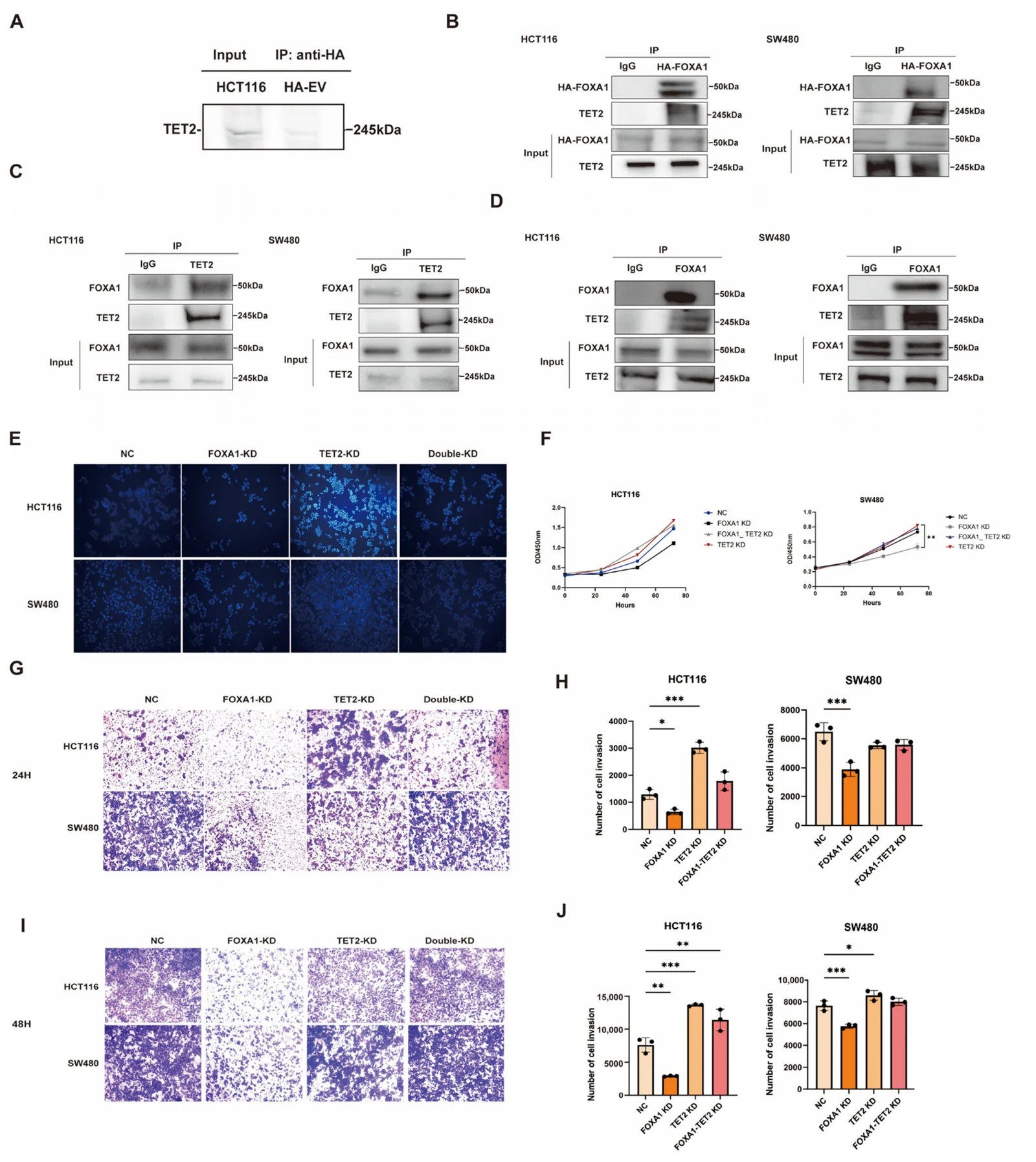
FOXA1 and TET2 protein-level associations and their effects on colorectal cancer cell growth-associated and invasion-associated phenotypes. (**A**) HA-tagged empty-vector control in HCT116 cells to evaluate nonspecific TET2 enrichment associated with the HA tag or the anti-HA immunoprecipitation system. (**B**) Exogenous co-immunoprecipitation analysis of HA–FOXA1 and TET2 in HCT116 and SW480 cells. (**C**) Endogenous TET2 immunoprecipitation followed by FOXA1 detection in HCT116 and SW480 cells. (**D**) Reciprocal endogenous FOXA1 immunoprecipitation followed by TET2 detection in HCT116 and SW480 cells. WB original images of (**B**–**D**) are included in Supplementary Figure S10. (**E**) CCK-8 analysis of HCT116 and SW480 cells after FOXA1 and/or TET2 knockdown at indicated time points (mean ± SD, *n* = 3). (**F**) Quantification of CCK-8 assay results. (**G**,**H**) Representative images and quantification of 24 h Matrigel Transwell invasion assays following FOXA1 and/or TET2 knockdown. (**I**,**J**) Representative images and quantification of 48 h Matrigel Transwell invasion assays following FOXA1 and/or TET2 knockdown. Data are presented as the mean ± SD from three independent experiments. Statistical comparisons are indicated in the corresponding graphs. * *p* < 0.05; ** *p* < 0.01; *** *p* < 0.001.

**Figure 6:**
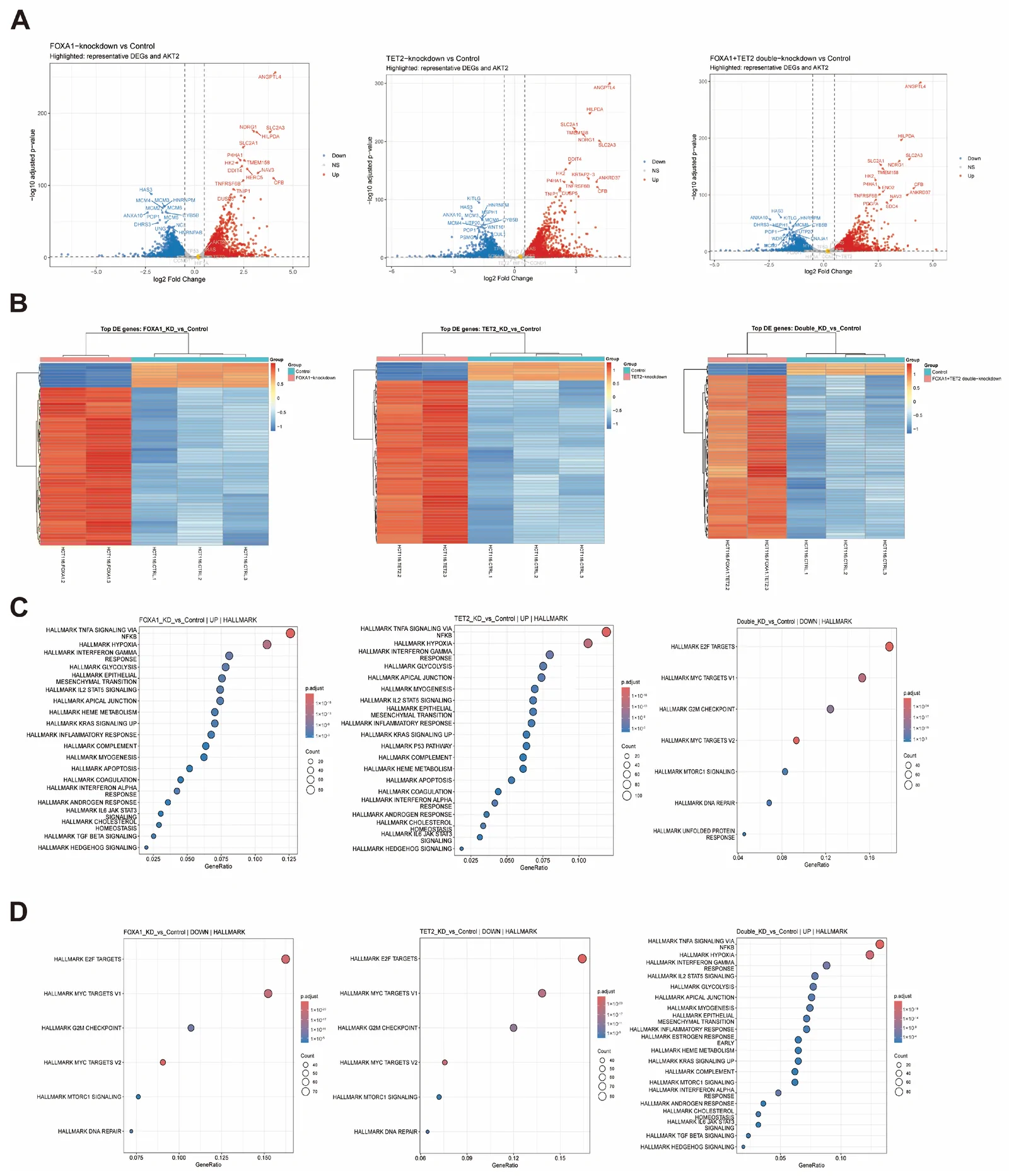
Differentially expressed genes and shared pathway alterations following FOXA1 and TET2 knockdown. (**A**) Volcano plots of differentially expressed genes in FOXA1-KD, TET2-KD, and Double-KD cells relative to the control group. The yellow dots indicate AKT2. **(B**) Z-score-normalized expression heatmaps of the top 60 differentially expressed genes under the three knockdown conditions. (**C**) Hallmark pathway enrichment analysis of genes upregulated following FOXA1, TET2, or combined FOXA1/TET2 knockdown. (**D**) Hallmark pathway enrichment analysis of genes downregulated following FOXA1, TET2, or combined FOXA1/TET2 knockdown. Pathways meeting the significance threshold are shown (all FDR < 0.05).

## Data Availability

The original contributions presented in this study are included in the article and its Supplementary Materials. Further inquiries can be directed at the corresponding authors.

## Supplementary Materials

The following supporting information can be downloaded at https://www.mdpi.com/article/10.3390/cancers18193110/s1, Figure S1: Supplementary analyses of FOXA1/TET2 genomic association and DNA methylation in TCGA-COAD; Figure S2: Screening and validation of candidate shRNAs targeting FOXA1 and TET2 in HEK293T cells; Figure S3: Quality assessment and representative gene expression profiles of RNA-seq data from control, FOXA1-KD, TET2-KD, and Double-KD HCT116 cells; Figure S4: Overlap of differentially expressed genes among FOXA1-KD, TET2-KD, and Double-KD HCT116 cells; Figure S5: Concordance of transcriptome-wide expression changes among FOXA1-KD, TET2-KD, and Double-KD HCT116 cells; Figure S6: Differentially expressed genes and pathway enrichment analysis specific to FOXA1 knockdown in HCT116 cells; Figure S7: Differentially expressed genes and pathway enrichment analysis specific to TET2 knockdown in HCT116 cells; Figure S8: corresponds to Figure 4C in the main manuscript and contains the full-membrane Western blot images for the FOXA1/TET2 knockdown experiments in HCT116 cells. Figure S9: corresponds to Figure 4D in the main manuscript and contains the full-membrane Western blot images for the FOXA1/TET2 knockdown experiments in SW480 cells. Figure S10: corresponds to Figure 5B–D in the main manuscript and contains the full-membrane images for the Co-IP experiments. Figure S11: contains the additional qRT-PCR and Western blot results requested by the reviewers, showing the changes in TET2 and FOXA1 expression following individual FOXA1 and TET2 knockdown, respectively. Table S1: Primer sequences used for RT–qPCR; Table S2: shRNA sequences targeting FOXA1 and TET2; Table S3: Antibodies used in this study.
