# Supplementary material for: Cross-Dataset Genomic Proximity and Functional Association of FOXA1 and TET2 at Hypomethylated Regulatory Regions in Colorectal Cancer
Source: Cancers (Basel). 2026 Sep 25;18(19):3110. doi: 10.3390/cancers18193110 (PMC13664142; doi:10.3390/cancers18193110)
Supplement: Supplementary file 1 [file cancers-18-03110-s001.zip › cancers-4485276-supplementary.pdf]

Supplementary Tables

Supplemental Table S1. Primer sequences used for RT-qPCR

Gene	Primer (5' - 3')
FOXA1-F	5'-GCCACTCGCTGTCCTTCAATG-3′
FOXA1-R	5'-CGCAAGTAGCAGCCGTTCTC-3′
TET2-F	5'-ACACAGCAACCCCAAACTGA-3′
TET2-R	5'-CATACAGGCATGTGGCTTGC-3′
18S rRNA-F	5'-GTCTGTGATGCCCTTAGATG-3′
18S rRNA-R	5'-AGCTTATGACCCGCACTTAC-3′

Supplemental Table S2. shRNA sequences targeting FOXA1 and TET2.

shRNA ID	Target Sequence (5' - 3')
FOXA1(human)-shRNA1	5′-GCGACTGGAACAGCTACTACG-3′
FOXA1(human)-shRNA2	5′-CCAGCGACTGGAACAGCTACT-3′
TET2(human)-shRNA1	5′-GGTGCTTACTTCAAGCAAA-3′
TET2(human)-shRNA2	5′-CGATTTGAGTGATAAGAAA-3′

Supplemental Table S3. Antibodies used in this study

Antibody	vendor	Catalog No.
FoxA1 Rabbit Monoclonal Antibody	Cell Signaling Technology (CST)	#53528
TET2 Rabbit Monoclonal Antibody	Cell Signaling Technology (CST)	#18950
Normal Rabbit IgG	Cell Signaling Technology (CST)	#2729
Anti-Mouse & Rabbit Light chain for IP	Hangzhou HuaAn Biotechnology Co., Ltd.	#001-180-005

Figure S1: Supplementary analyses of FOXA1/TET2 genomic association and DNA methylation in TCGA-COAD;

Figure S2: Screening and validation of candidate shRNAs targeting FOXA1 and TET2 in HEK293T cells;

Figure S3: Quality assessment and representative gene expression profiles of RNA-seq data from Control, FOXA1-KD, TET2-KD, and Double-KD HCT116 cells;

Figure S4: Overlap of differentially expressed genes among FOXA1-KD, TET2-KD, and Double-KD HCT116 cells;

Figure S5: Concordance of transcriptome-wide expression changes among FOXA1-KD, TET2-KD, and Double-KD HCT116 cells;

Figure S6: Differentially expressed genes and pathway enrichment analysis specific to FOXA1 knockdown in HCT116 cells;

Figure S7: Differentially expressed genes and pathway enrichment analysis specific to TET2 knockdown in HCT116 cells;

Figure S8: corresponds to Figure 4C in the main manuscript and contains the full-membrane Western blot images for the FOXA1/TET2 knockdown experiments in HCT116 cells.

Figure S9: corresponds to Figure 4D in the main manuscript and contains the full-membrane Western blot images for the FOXA1/TET2 knockdown experiments in SW480 cells.

Figure S10: corresponds to Figure 5B–D in the main manuscript and contains the full-membrane images for the Co-IP experiments.

Figure S11: contains the additional qRT-PCR and Western blot results requested by the reviewers, showing the changes in TET2 and FOXA1 expression following individual FOXA1 and TET2 knockdown, respectively.
